# MicroRNA-16-5p overexpression suppresses proliferation and invasion as well as triggers apoptosis by targeting VEGFA expression in breast carcinoma

**DOI:** 10.18632/oncotarget.20398

**Published:** 2017-08-23

**Authors:** Yunhui Qu, Hongtao Liu, Xinquan Lv, Yuqiong Liu, Xiaojuan Wang, Min Zhang, Xiaqing Zhang, Yuenan Li, Qianqian Lou, Shenglei Li, Huixiang Li

**Affiliations:** ^1^ Department of Pathology, School of Basic Medical Sciences, Zhengzhou University and The First Affiliated Hospital of Zhengzhou University, Zhengzhou, 450001, Henan, P.R. China; ^2^ Clinical Laboratory Medicine, The First Affiliated Hospital of Zhengzhou University, Zhengzhou, 450001, Henan, P.R. China; ^3^ Laboratory for Cell Biology, College of Life Sciences of Zhengzhou University, Zhengzhou, 450001, Henan, P.R. China

**Keywords:** microRNA-16-5p, breast carcinoma, HIF-α, VEGFA, tumor growth

## Abstract

MicroRNAs (miRNAs), a class of small noncoding RNA molecules, can manipulate the expressions of endogenous tumor-related genes, and are implicated in the development and progression of a wide type of tumors. In this study, the investigation from real-time quantitative PCR revealed that miRNA-16-5p was downregulated in breast carcinoma tissues and cells, coupled with the elevations of HIF-α and VEGFA protein expressions, compared with normal tissues. Lentiviral armed with miR-16-5p markedly increased the miR-16-5p levels in MCF-7 and MDA-MB-231 cells, compared to blank and NC groups, and miR-16-5p overexpression significantly inhibited the proliferation and colony formation in MCF-7 and MDA-MB-231 cells. Besides, miR-16-5p upregulation markedly induced apoptosis and reduced invasion ability in MCF-7 and MDA-MB-231 cells. Notably, VEGFA was direct target of miR-16-5p. Stepwise investigation from *in vitro* and *in vivo* experiments demonstrated that miR-16-5p overexpression suppressed tumor growth and reduced HIF-α and VEGFA expressions in breast carcinoma cells and nude mice tumor tissues. These findings provide novel insights into molecular mechanism involved in the roles of miR-16-5p in tumor development and progression of breast carcinoma, and thus manipulation of miR-16-5p may be a novel potential therapeutic target for future therapies of the patients with breast carcinoma.

## INTRODUCTION

Breast carcinoma, a kind of highly heterogeneous disease characterized by a wide variety of molecular and pathologic diversity [1, 2], is one of the most frequently diagnosed women tumor types worldwide [3]. Based on the data from the WHO World Cancer Report in 2015, there are approximately 14 million patients diagnosed newly and 8.2 million deaths in the world [4]. The process of development and progression of breast carcinoma was triggered by many factors, such as lifestyle, environmental, genetic and reproductive, etc [5–7]. More than 90% of the patients with breast carcinoma died due to metastasis [8]. These features will lead to the results that the different patients with breast carcinoma have different prognosis and response to tumor therapies [9]. Therefore, it is imperative to seek for novel molecular target for the diagnosis and therapy of the patients with breast carcinoma.

Small non-coding RNAs, a class of highly conserved molecules, play important roles in the regulation of gene expression, cell cycle, apoptosis, invasion, metastasis, and signaling pathways in a variety of tumors [10]. MicroRNAs (miRNAs) belong to short non-coding RNAs comprising of 21–25 nucleotides, and has been verified to be closely associated with mRNA degradation, transcriptional repression, and tumor microenvironment [11–13], and may be a novel molecular target for tumor patients [14–16]. Our current investigation revealed that miR-16-5p was downregulated in breast carcinoma in 74 cases of breast carcinoma tissues and paired normal breast tissues by real-time quantitative PCR, which will impel us to further investigate the biological function of miR-16-5p in the development and progression of breast carcinoma, which was not so far reported in the world.

Therefore, in the current study, we investigated the roles of miR-16-5p in the development and progression of breast carcinoma from *in vitro* and *in vivo* experiments, including tumor growth *in vitro* and *in vivo*, cell apoptosis and cell invasion ability. Most notably, our current results confirmed miR-16-5p mediated biology function may be tightly related to HIF-α and VEGFA expressions, which was directly correlated with tumor development and progression, and thus manipulation of miR-16-5p may be a novel molecular target for the patients with breast carcinoma.

## RESULTS

### Reduced miR-16-5p as well as HIF-α and VEGFA levels in breast carcinoma

**Figure 1:**
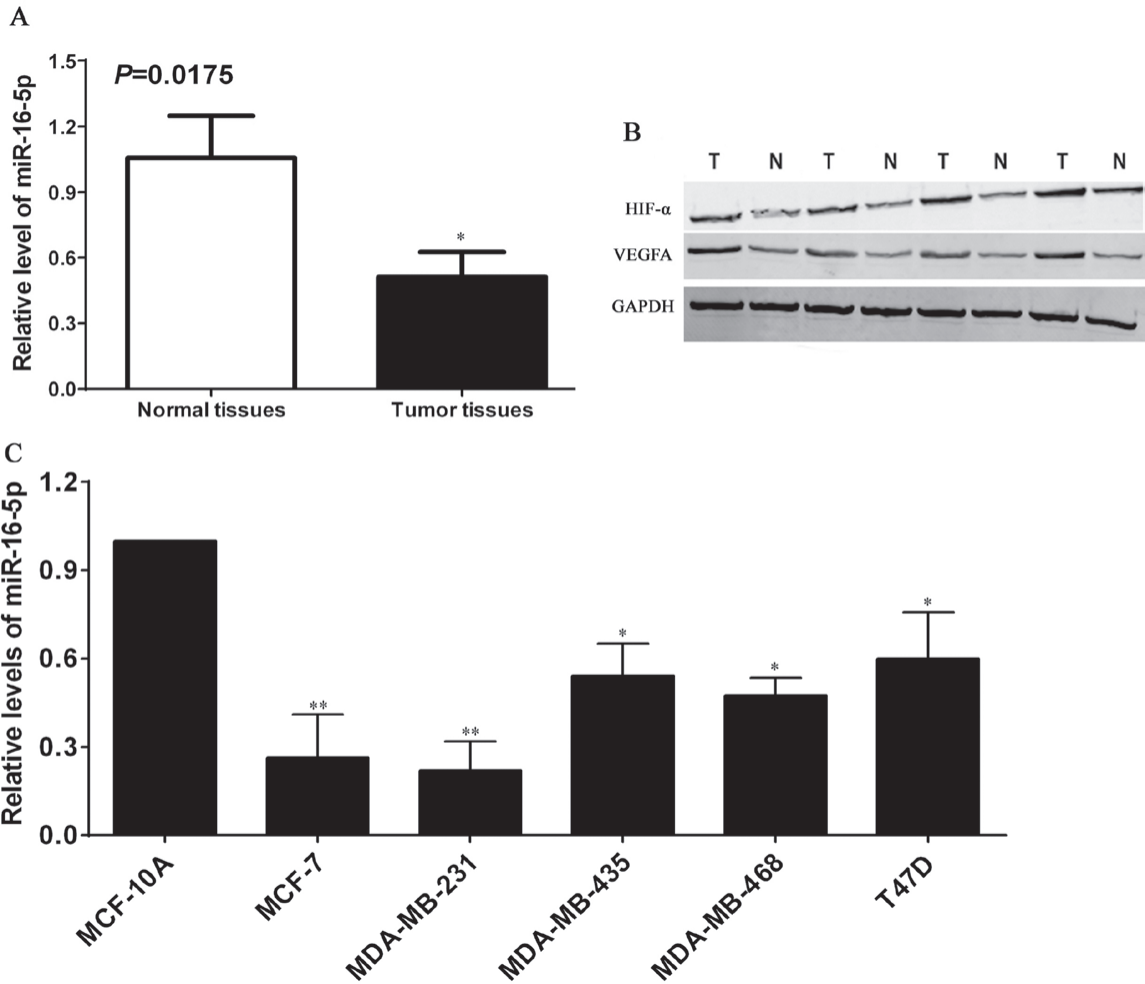
Expression profiles of miR-16-5p as well as HIF-α and VEGFA proteins in breast carcinoma 74 cases of breast carcinoma tissues and corresponding normal breast tissues were collected from the First Affiliated Hospital of Zhengzhou University, total RNA and proteins were extracted from the tissues above, and Real-time quantitative PCR and Western blotting were used to detect the miR-16-5p as well as HIF-α and VEGFA protein expression, respectively. (**A**) Relative expression assay of miR-16-5p in breast carcinoma tissues and paired normal breast tissues, *P < 0.05, compared with normal breast tissues. (**B**) Expressions of HIF-α and VEGFA proteins in breast carcinoma tissues (T) and normal breast tissues (N), GAPDH was used as an internal control. (**C**) Relative expression assay of miR-16-5p in breast carcinoma cells (MCF-7, MDA-MB-231, MDA-MB-435, MDA-MB-468 and T47D) and benign non-tumorigenic MCF10A cells; *P < 0.05, compared with MCF-10A cells; **P < 0.01, compared with the other cells.

To confirm the roles of miR-16-5p in the development and progression of breast carcinoma, Real-time quantitative PCR was utilized to detect the expression of miR-16-5p in breast carcinoma tissues. We found that the expression of miR-16-5p in breast carcinoma tissues was significantly lower than that in paired normal tissues (*P* < 0.05) (Figure 1A). Converse results from Western blot were found regarding the expressions of HIF-α and VEGFA proteins (Figure 1B). Stepwise investigation revealed that the expression of miR-16-5p in breast carcinoma cells were significantly lower than that in benign non-tumorigenic MCF10A cells (*P* < 0.05) (Figure 1C), in which MCF-7 and MDA-MB-231 exhibited lowest endogenous miR-16-5p level (*P* < 0.01) (Figure 1C). These data support that miR-16-5p functions as tumor suppressor in the development and progression of breast carcinoma.

### Lentiviral vector carrying miR-16-5p significantly increased miR-16-5p level in breast carcinoma cells

**Figure 2:**
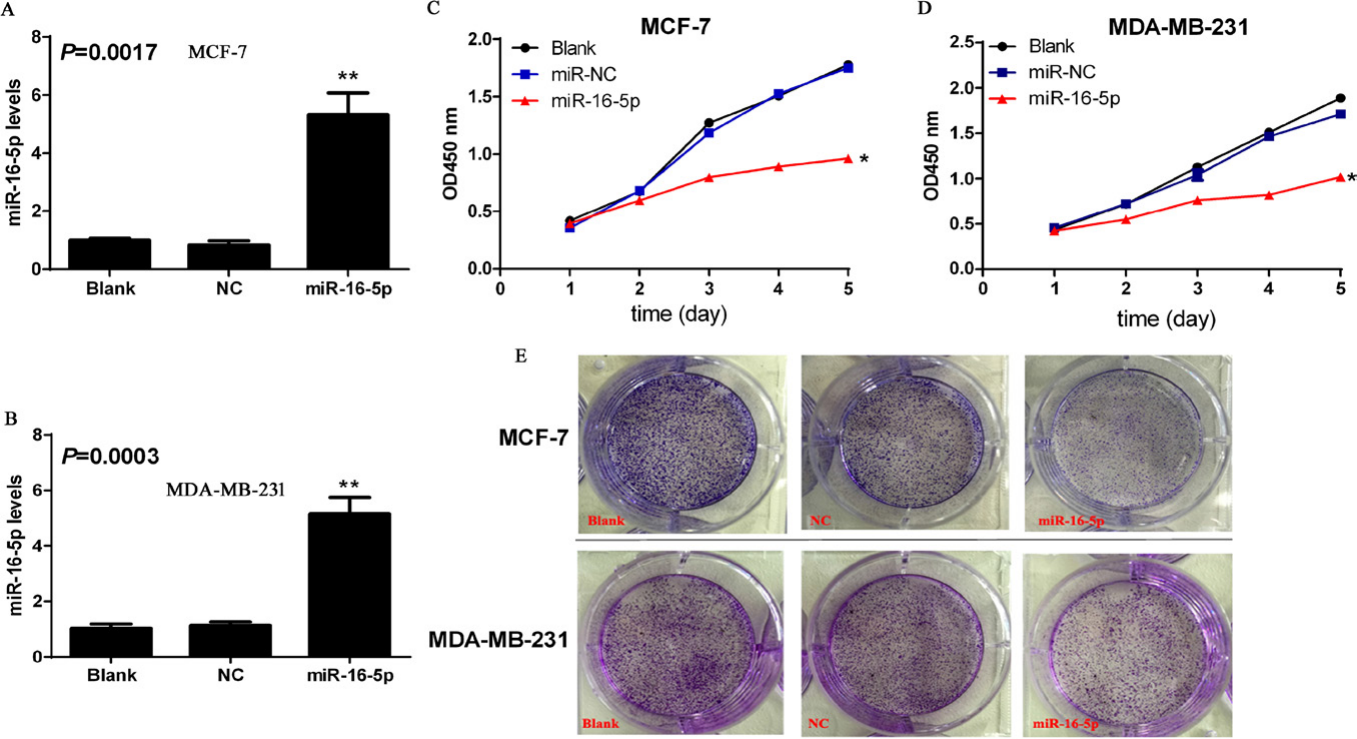
Overexpression of miR-16-5p contributed to the suppresses of cell proliferation and colony formation in MCF-7 and MDA-MB-231 cells Lentiviral vector armed with miR-16-5p and control empty vector were transfected to breast carcinoma cell lines MCF-7 and MDA-MB-231 cells, and stable clones were selected using G418. Subsequently, Real-time quantitative PCR was used to investigate the relative levels of miR-16-5p in different treatment cells including MCF-7 and MDA-MB-231. (**A**) Relative level of miR-16-5p in different treatment MCF-7 cells, **P < 0.01, compared with blank group and NC group. (**B**) Relative level of miR-16-5p in different treatment MDA-MB-231 cells, **P < 0.01, compared with blank group and NC group. (**C**) Overexpression of miR-16-5p significantly inhibited the proliferation of MCF-7 cells. Cells with different treatments (2000/well) were seeded into 96-well plates, CCK-8 kits were was added to corresponding wells and absorbance value at 450 nm were determined using microplate reader at different time points including days 1, 2, 3, 4 and 5. (**D**) Overexpression of miR-16-5p significantly inhibited the proliferation of MDA-MB-231 cells. Cells with different treatments (2000/well) were seeded into 96-well plates, CCK-8 kits were was added to corresponding wells and absorbance value at 450 nm were determined using microplate reader at different time points including days 1, 2, 3, 4 and 5. (**E**) Overexpression of miR-16-5p markedly inhibited colony formation in MCF-7 and MDA-MB-231 cells. Cells with different treatment were seeded into soft agar culture, and the pictures were taken on day 12 using microscopy picture system.

To investigate the function of miR-16-5p in breast carcinoma, lentiviral vector carrying miR-16-5p and control vector were transfected to breast carcinoma cells, and real-time quantitative PCR was employed to determine the expression of miR-16-5p in MCF-7 and MDA-MB-231 cells. We found that lentiviral armed with miR-16-5p markedly increased the miR-16-5p levels in MCF-7 and MDA-MB-231 cells, compared to blank and NC groups (*P* < 0.05) (Figure 2A and 2B). These findings will provide the theoretical basis for further investigation of the function of miR-16-5p in breast carcinoma.

### MiR-16-5p overexpression contributed to the inhibition of proliferation and colony formation ability in breast carcinoma cells

To verify the roles of miR-16-5p in the regulation of proliferation and colony formation ability in breast carcinoma cells, CCK-8 and soft agar colony formation experiments were used to investigate the potential roles of miR-16-5p in the proliferation and colony formation ability of breast carcinoma. The results demonstrated that miR-16-5p overexpression significantly suppressed cell proliferation and colony formation ability both in MCF-7 cells and MDA-MB-231 cells, compared with blank and NC groups (Figure 2C–2E). Therefore, miR-16-5p may be a potential molecular target for breast carcinoma.

### Overexpression of miR-16-5p induced apoptosis in breast carcinoma cells

**Figure 3:**
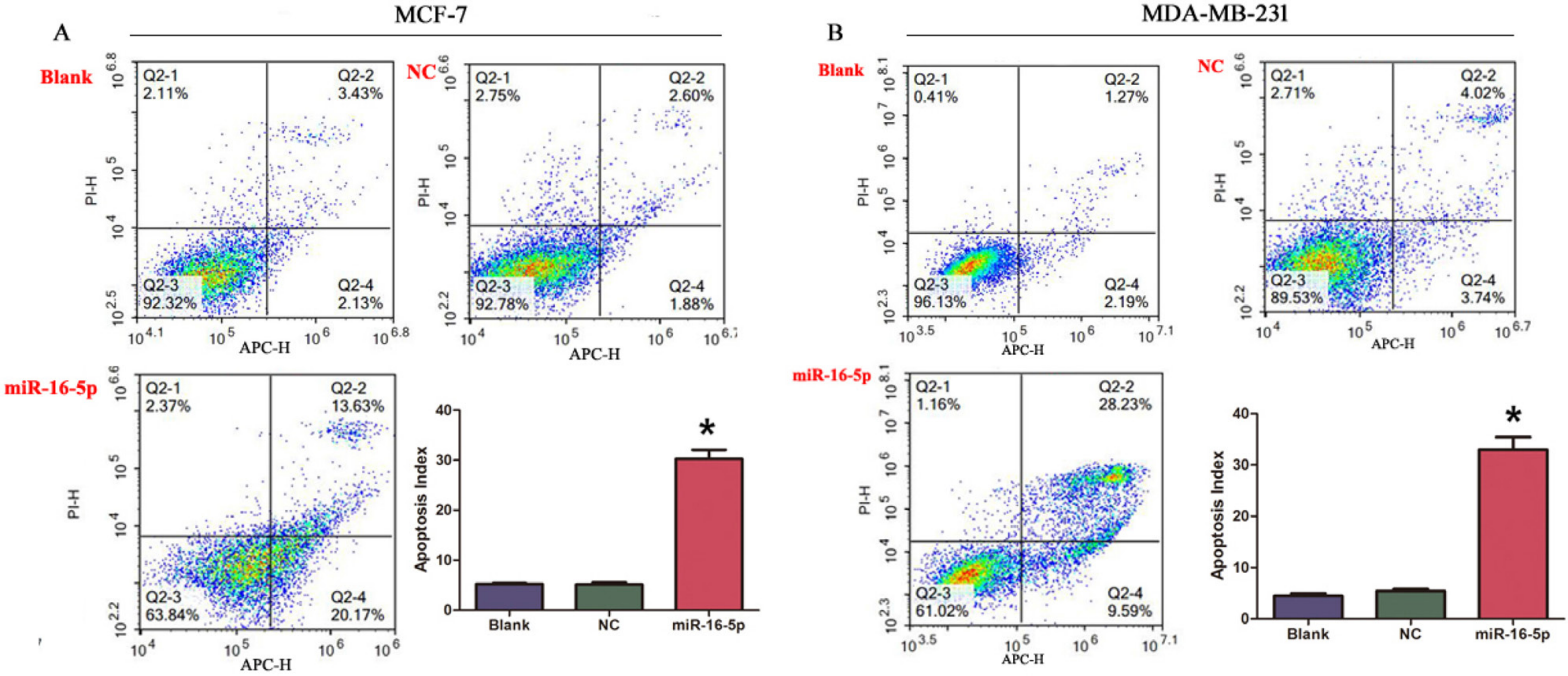
Overexpression of miR-16-5p induced cell apoptosis in MCF-7 cells and MDA-MB-231 cells (**A**) miR-16-5p induced apoptosis of MCF-7 cells. (**B**) miR-16-5p induced apoptosis of MDA-MB-231 cells. *P < 0.05, compared with blank group and NC group.

In this study, we performed cell apoptosis assay in different treatment MCF-7 cells and MDA-MB-231 cells. The current results revealed that overexpression of miR-16-5p markedly induced apoptosis in MCF-7 and MDA-MB-231 cells, compared with blank and NC groups (Figure 3A and 3B). These findings suggest the essential role of miR-16-5p in the apoptosis of breast carcinoma cells.

### Overexpression of miR-16-5p reduced cell invasion ability in breast carcinoma cells

**Figure 4:**
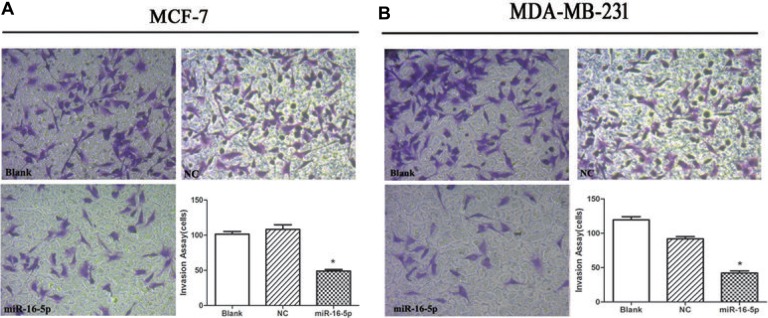
Overexpression of miR-16-5p suppressed cell invasion ability in MCF-7 and MDA-MB-231 cells 5 × 10E4 of MCF-7 cells and MDA-MB-231 cells were seeded into transwell chamber. Invading cells was taken using microscopy system at 48 h. *P < 0.05, compared with blank group and NC group.

To further confirm the role of miR-16-5p in cell invasion ability in breast carcinoma, transwell chamber was used to detect cell invasion ability in different treatment MCF-7 and MDA-MB-231 cells. We found that the invasive cell numbers in miR-16-5p overexpression group was significantly lower than those in blank and NC groups (*P* < 0.05) (Figure 4A and 4B).

### VEGFA as a direct target of miR-16-5p

**Figure 5:**
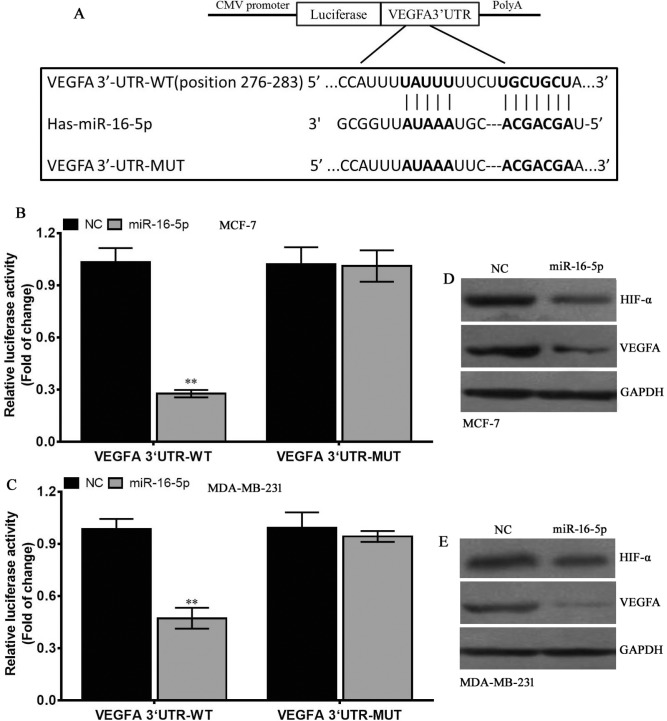
VEGFA is a direct target of miR-16-5p (**A**) The VEGFA 3′-UTR-WT or –MUT was inserted into the downstream of luciferase reporter vector. The mutated sequences were in bold. (**B**) The luciferase activity was controlled by VEGFA 3′-UTR by ectopic miR-16-5p expression in MCF-7 cells. **P < 0.01, compared to NC group. (**C**) The luciferase activity was controlled by VEGFA 3′-UTR by ectopic miR-16-5p expression in MDA-MB-231 cells. **P < 0.01, compared to NC group. (**D**) Western blot assay for HIF-α and VEGFA protein expressions in different treatment MCF-7 cells, and GAPDH was used as a loading control. (**E**) Western blot assay for HIF-α and VEGFA protein expressions in different treatment MDA-MB-231 cells, and GAPDH was used as a loading control.

To further elucidate the molecular mechanisms mediated by miR-16-5p, TargetScan, MicroRNA.ORG and miRDB were used to investigate the potential target of miR-16-5p, and preliminarily confirmed VEGFA was a potential target of miR-16-5p (Figure 5A). To verify whether VEGFA was the direct target of miR-16-5p, VEGFA-3′-UTR-WT and VEGFA-3′-UTR-MUT were constructed (Figure 5A). These vectors, along with pRL-SV40 were co-transfected into breast carcinoma MCF-7 and MDA-MB-231 cells with LV1-miR-16-5p or LV1-NC, and the luciferase activity was measured. The results revealed that miR-16-5p significantly reduced the luciferase activity of VEGFA-3′-UTR-WT, but didn't affect that of VEGFA-3′-UTR-MUT in MCF-7 and MDA-MB-231 cells (Figure 5B and 5C), implying miR-16-5p directly binds to the 3′-UTR region of VEGFA. Further investigation demonstrated that miR-16-5p overexpression reduced VEGFA and HIF-α protein levels in MCF-7 and MDA-MB-231 (Figure 5D and 5E). These data suggest that miR-16-5p suppresses VEGFA protein expression by directing binding to its 3′-UTR region.

### Overexpression of miR-16-5p significantly suppressed tumor growth in MCF-7 and MDA-MB-231 xenografts

**Figure 6:**
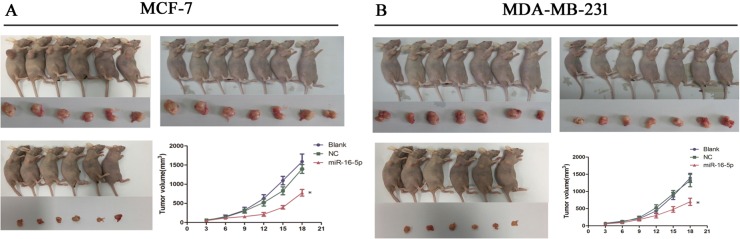
miR-16-5p significantly suppressed tumor growth in MCF-7 and MDA-MB-231 xenografted tumors MCF-7 cells and MDA-MB-231 at a density of 5 × 10e6 with different treatments were subcutaneously injected into the back of nude mice, and tumor volume was measured twice every week. Finally, tumor growth curve was made to determine the effect of miR-16-5p expression on tumor growth. (**A**) miR-16-5p overexpression markedly inhibited tumor growth in MCF-7 xenografted tumors; (**B**) miR-16-5p overexpression markedly inhibited tumor growth in MDA-MB-231 xenografted tumors. *P < 0.05, compared with blank group and NC group.

To explore the antitumor efficacy of miR-16-5p in breast carcinoma, MCF-7 and MDA-MB-231 exnografts were established by injecting different doses MCF-7 and MDA-MB-231 cell numbers with different treatment. We found that miR-16-15 significantly inhibited tumor growth, compared with blank group and NC group, in MCF-7 and MDA-MB-231 xenografts (Figure 6A and 6B). *In vivo* experiment suggests that miR-16-5p may be a novel tumor molecular target for breast carcinoma.

### Overexpression of miR-16-5p reduced HIF-α and VEGFA expression in MCF-7 and MDA-MB-231 xenografts

**Figure 7:**
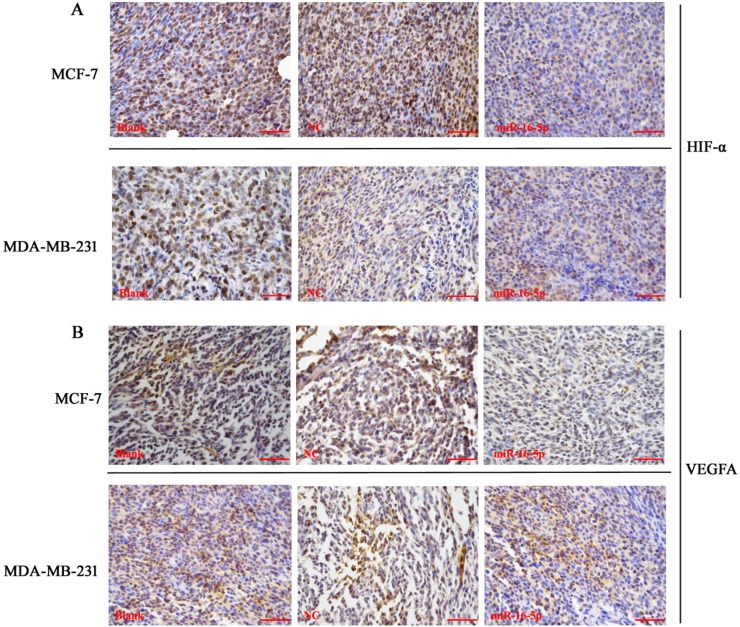
Immunohistochemistry assay for HIF-α and VEGFA protein expression in different treatment nude mice tumor tissues When the measurement was terminated, tumor tissues were obtained, immunohistochemistry was employed to detect the HIF-α and VEGFA protein expression using corresponding primary antibody. (**A**) Immunohistochemistry assay for HIF-α protein expression in MCF-7 and MDA-MB-231 cells with different treatments; (**B**) Immunohistochemistry assay for VEGFA protein expression in MCF-7 and MDA-MB-231 cells with different treatments, Bar = 100 μm

**Figure 8:**
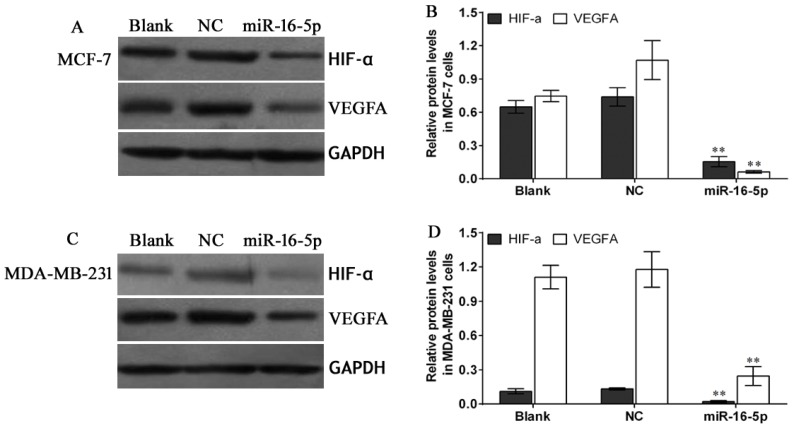
Western blot assay for HIF-α and VEGFA protein expression in different treatment nude mice tumor tissues When the measurement was terminated, tumor tissues were obtained, Western blot was employed to detect the HIF-α and VEGFA protein expression using corresponding primary antibodies against HIF-α and VEGFA protein. (**A**) Western blot assay for HIF-α and VEGFA protein expressions in MCF-7 cells with different treatments, and GAPDH was used as a loading control; (**B**) Statistical assay for HIF-α and VEGFA protein expressions in different treatment MCF-7 cells xenografted nude mice tumor tissues, **P < 0.01, compared with blank group and NC group; (**C**) Western blot assay for HIF-α and VEGFA protein expressions in MDA-MB-231 cells with different treatments, and GAPDH was used as a loading control; (**D**) Statistical assay for HIF-α and VEGFA protein expressions in different treatment MDA-MB-231 cells xenografted nude mice tumor tissues, **P < 0.01, compared with blank group and NC group

To verify the relationship between miR-16-5p and HIF-α as well as VEGFA expressions in breast carcinoma, immunohistochemistry and Western blot were employed to investigate the HIF-α and VEGFA expressions in nude mice tumor tissues. The results showed that expressions of HIF-α and VEGFA proteins in miR-16-5p treatment groups were obviously lower than those in blank and NC groups; however, there were no differences in expressions of HIF-α and VEGFA proteins between blank group and NC group (Figure 7A and 7B, Figure 8A–8D). These findings suggest that antitumour efficacy of miR-16-5p in breast carcinoma may be partly achieved by reducing HIF-α and VEGFA expressions.

## DISCUSSION

More and more evidence has demonstrated that miRNAs have a wide variety of biological functions mainly implicated in several cell signaling pathways essential to tumor development and progression, including proliferation, apoptosis, differentiation, invasion and metastasis [18, 19], which mainly function either as oncogenes or tumor suppressor genes [20, 21], which will open up new opportunities for a large number of tumors. Many studies have revealed that differential miRNA expression is a common event in many tumors in expression data from microRNA array [22–25]. In this study, real-time quantitative PCR was used to investigate the miR-16-5p in 74 cases breast carcinoma and matched normal tissues, we found miR-16-5p exhibited lower expression in breast carcinoma tissues than in normal breast tissues, which was supported by the results from different breast carcinoma cells. These findings suggest that miR-16-5p may be tightly associated with the development and progression of breast carcinoma, and thus miR-16-5p will provide a novel molecular target for therapy of the patients with breast carcinoma.

Investigation from the other groups showed that miR-16-5p expression was exhibited at similar levels in most tissues [26], and was recommended as an internal control in breast tissues or serum and plasma [27–30]. These findings are diametrically opposed to our results, which will further force us to investigate the roles of miR-16-5p in breast carcinoma. In addition, a large amount of studies involved in miRNAs showed miRNA participated in the occurrence and development of many tumors, and thus inhibition or overexpression of miRNAs will be novel therapy strategies for tumor growth. Zhang J, et al. found miR-16-5p was downregulated in gastric carcinoma [31], which was consistent with our results in breast carcinoma. However, miR-16-5p was significantly upregulated in Kaposi's sarcoma [32]. These results reflect the facts that miR-16-5p level may depend on tumor types, and further exerts different biological role in various tumors. To verify whether miR-16-5p overexpression contributes to growth inhibition of breast carcinoma, lentiviral vector armed with miR-16-5p was used to transfect breast carcinoma cell lines, we found lentiviral vector carrying miR-16-5p significantly increased miR-16-5p level in breast carcinoma cells, and further investigation found miR-16-5p overexpression markedly inhibited tumor growth *in vitro* and *in vivo*, suggesting miR-16-5p may be a potential molecular target for the patients with breast carcinoma.

Resisting cell death and activating invasion and metastasis are two main hallmarks of tumors [33]. Many miRNAs are implicated in the process of cell apoptosis, invasion and metastasis. Liu Z, et al. found that inhibition of miR-221 expression obviously induced apoptosis and inhibited growth and invasion in Hepatocellular carcinoma HepG2 cells [34]. In addition, miR-150 overexpression inhibited invasion and metastasis of osteosarcoma cells, which was achieved by decreasing Ezrin expression [35]. These findings imply that miRNAs play an important role in the regulation of cell apoptosis as well as invasion and metastasis in a wide variety of tumors. To further verify the roles of miR-16-5p in cell apoptosis as well as invasion of breast carcinoma, we found that miR-16-5p overexpression significantly induced cell apoptosis, meanwhile, reduced invasion ability in breast carcinoma cells. Further bioinformation assay revealed that VEGFA may be the potential target gene of miR-16-5p. Stepwise investigation demonstrated miR-29c directly bound to the 3′-UTR region of VEGFA, and reduced VEGFA protein expression in breast carcinoma cells, coupled with the downregulation of HIF-α. To deeply understand the molecular mechanisms of miR-16-5p *in vivo*, we found that miR-16-5p overexpression evidently downregulated HIF-α and VEGF protein expression in nude mice tumor tissues, which were an accepted fact that these two proteins play an essential role in the development and progression of many tumors by multiple different mechanisms [36–46]. These findings highlight the potential therapeutic value of miR-16-5p in breast carcinoma, and combination of miR-16-5p with the related signaling pathway of HIF-α and VEGFA may be an effective molecular target for the patients with breast carcinoma in future.

In conclusion, our current study show miR-16-5p is significantly downregulated in breast carcinoma, and its overexpression contributes to growth inhibition *in vitro* and *in vivo*, cell apoptosis and the decrease of invasion ability, which is at least in part achieved by directly targeting VEGFA. These findings suggest that miR-16-5p may be a potential molecular target for the patients with breast carcinoma. More detailed and new insights into molecular mechanisms of miR-16-5p in the development and progression of breast carcinoma are urgently needed to be elucidated, which will build a solid foundation for the clinic transformation of miR-16-5p in the future.

## MATERIALS AND METHODS

### Tissue samples

Breast carcinoma tissues and matched normal tissues were obtained from the First Affiliated Hospital of Zhengzhou University, Zhengzhou, Henan Province, China. All samples was consented with informal written, and wasn't received any treatments prior surgery, including radiotherapy, chemotherapy and immune therapy. The tissues were used to detect microRNA expression using real-time quantitative PCR. The current study was approved by the Institutional Research Ethics Committee of Zhengzhou University.

### Cell culture

Breast carcinoma cell lines including MCF-7 and MDA-MB-231 cells were provided by Professor Yaohe Wang (Cell and Gene Therapy Research Centre, the Academy of Medical Science, Zhengzhou University), the other breast carcinoma cells including MDA-MB-435, MDA-MB-468 and T47D as well as benign non-tumorigenic MCF10A cells were obtained directly from the ATCC (Manassas, VA, USA). The cell lines above were maintained in RPMI-1640 culture supplemented with 10% fetal bovine serum (FBS) (Sigma-Aldrich, USA), 100 U/ml penicillin (Sigma-Aldrich, USA) and 100μg/ml streptomycin (Sigma-Aldrich, USA). Breast carcinoma cell lines above were maintained in 37°C with 5% CO_2_ in a incutabor.

### Lentiviral vector and transfection

LV1-miR-16-5p and LV1-NC were both constructed by Genepharma company (Shanghai, China), packaged in 293T cells, and measured virus titers for 10^8^ TU/ml. MCF-7 and MDA-MB-231 cells were infected using LV1-miR-16-5p and LV1-NC viruses, and clones stably expressing miR-16-5p and NC were selected using puromycin (Sigma-Aldrich, USA) according to manufacturer's instructions.

### Bioinformatics assay

The downstream target genes of miR-16-5p were predicted by three online programs with different databases involved in various algorithms, such as TargetScan (http://www.targetscan.org/), miRDB (http://mirdb.org/) and microRNA.org (http://www.microrna.org/).

### Plasmid construction and luciferase reporter assay

To construct VEGFA-3′-UTR-wild type (VEGFA-3′-UTR-WT) vector, human VEGFA 3′-UTR region with miR-16-5p binding sequences was amplified, which was ligated to the pGV126 vector (GeneChem, China), VEGFA-3′-UTR-mutation (VEGFA-3′-UTR-MUT) vector with a substitution of 12 bp in miR-16-5p binding region. The 3′-UTR region in the two vectors was inserted into the downstream region of firefly luciferase gene. MCF-7 and MDA-MB-231 cells were co-transfected using reporter plasmids (400 ng per 20 ng internal control renilla luciferase plasmid pRL-SV40) and LV1-miR-16-5p or LV1-NC by Lipofectamine 2000 (Invitrogen, USA). Subsequenly, cells were harvested at 48 h after transfection with plasmids above, and then was investigated using the Dual Luciferase Assay Kit (Promega, USA) by Synergy H1 hybrid reader (Biotek, USA). Finally, luciferase activity was normalized to the renilla luciferase activity.

### Real-time quantitative PCR

Total RNA was isolated from tissues and cells, which was subjected to the first strand cDNA kit (Sangon Bioech, Shanghai, China). Real-time quantitative PCR (Takara, Dalian, China) was used to detect the miR-16-5p expression in StepOne Plus PCR instruments using SYBR Green kit (Tiangen Biotech, Beijing, China).

### Western blot

Total proteins were extracted from breast carcinoma tissues, paired normal tissues, and different treatment breast carcinoma cells by RIPA and PMSF (Solarbio, Beijing, China). Protein concentrations were determined using Bradford methods according to manufacturer's protocol (Solarbio, Beijing, China). Subsequently, SDS-PAGE was performed followed by electro-transferred to PVDF membrane (Sigma-Aldrich, USA). After blocking with skimmed milk, primary antibodies against HIF-α(CST, 14179S, 120 KDa, USA) and VEGFA (Abcam, ab46154, 15-40 KDa, USA) were incubated with PVDF membrane (Roche, Switzerland) overnight at room temperature. The second antibody (LI-COR, C60405-05, USA) was added to PVDF membrane after termination of primary incubation. Finally, signal of protein expression was developed using Licor Odyssey (LI-COR, USA).

### CCK-8 determination for cell proliferation

Different cell lines including MCF-7 and MDA-MB-231 cells with different treatments at a density of 2000 cells/well were seeded into 96-well plate. Cell proliferation was measured according to manufacturer's protocol. At the time of measuring cell proliferation, CCK-8 reagent (Dojindo, Japan) was added to corresponding well for continuous culture for 3 h; finally, absorbance values at 450 nm were determined using microplate reader.

### Cell apoptosis assay

Cell apoptosis was performed using standard Annexin V/PI staining by Flow cytometry. MCF-7 and MDA-MB-231 cells were collected using trypsinase, and Annexin V/PI (Sigma-Aldrich, USA) reagents were added to EP tube for 30 min. Finally, Flow cytometry (BD Biosciences) was used to determine cell apoptosis in different treatment groups.

### Cell invasion experiment

Cell invasion experiment was performed using Transwell chamber with Matrigel (BD Company). Briefly, MCF-7 and MDA-MB-231 cells with different treatments (1 × 10E5) was added to upper layer of chamber, and 20% FBS was added to underlayer of chamber. At 48 h, invading cells were fixed using methanol and stained with crystal violet. Finally, invading cell numbers were counted under the field of 200× magnification.

### Tumor growth *in vivo*

Different treatment MCF-7 and MDA-MB-231 cells were subcutaneously injected into the back of nude mice. Tumor volumes were measured twice every week. Tumor growth curve was made to determine the effects of miR-16-5p on tumor growth.

### Immunohistochemistry

Immunohistochemistry was performed according to previous reports [17]. In brief, tissue slides were fixed using formalin, embedded in paraffin and cut continuously for 4–6 μm for further immunohistochemistry assay. After deparaffinization, rehydration and pretreatment using microwave heating in citrate buffer (pH 6.0). Primary antibodies against HIF-α and VEGFA were incubated with tissue sections, after rinsing, the corresponding second antibody was added to tissue slides. Staining signals were developed using DAB reagent. The staining results were evaluated by two excellent pathologists.

### Statistical treatment

Statistical assay was performed using SPSS17.0 software. Data were expressed as means ± SD, which were from at least three times independently repeats. The comparisons of two groups were investigated using *t* test, and comparisons of three groups or above were analyzed using One way ANOVA. A *P* value less than 0.5 was considered as significant difference.
